# Multiple graphical views for automatically generating SQL for the MycoDiversity DB; making fungal biodiversity studies accessible

**DOI:** 10.3897/BDJ.12.e119660

**Published:** 2024-06-18

**Authors:** Irene Martorelli, Aram Pooryousefi, Haike van Thiel, Floris J Sicking, Guus J Ramackers, Vincent Merckx, Fons J Verbeek

**Affiliations:** 1 Leiden Institute of Advanced Computer Science (LIACS), Leiden University, Leiden, Netherlands Leiden Institute of Advanced Computer Science (LIACS), Leiden University Leiden Netherlands; 2 Naturalis Biodiversity Center, Leiden, Netherlands Naturalis Biodiversity Center Leiden Netherlands; 3 Institute for Biodiversity and Ecosystem Dynamics, University of Amsterdam, Amsterdam, Netherlands Institute for Biodiversity and Ecosystem Dynamics, University of Amsterdam Amsterdam Netherlands

**Keywords:** fungal distribution, fungal biodiversity, biogeography, environmental DNA, mycodiversity, geospatial maps, information visualisation, dynamic hierarchical data, accessibility, reusability, database, FAIR data, controlled vocabulary terms

## Abstract

Fungi is a highly diverse group of eukaryotic organisms that live under an extremely wide range of environmental conditions. Nowadays, there is a fundamental focus on observing how biodiversity varies on different spatial scales, in addition to understanding the environmental factors which drive fungal biodiversity. Metabarcoding is a high-throughput DNA sequencing technology that has positively contributed to observing fungal communities in environments. While the DNA sequencing data generated from metabarcoding studies are available in public archives, this valuable data resource is not directly usable for fungal biodiversity investigation. Additionally, due to its fragmented storage and distributed nature, it is not immediately accessible through a single user interface. We developed the MycoDiversity DataBase User Interface (https://mycodiversity.liacs.nl) to provide direct access and retrieval of fungal data that was previously inaccessible in the public domain. The user interface provides multiple graphical views of the data components used to reveal fungal biodiversity. These components include reliable geo-location terms, the reference taxonomic scientific names associated with fungal species and the standard features describing the environment where they occur. Direct observation of the public DNA sequencing data in association with fungi is accessible through SQL search queries created by interactively manipulating topological maps and dynamic hierarchical tree views. The search results are presented in configurable data table views that can be downloaded for further use. With the MycoDiversity DataBase User Interface, we make fungal biodiversity data accessible, assisting researchers and other stakeholders in using metabarcoding studies for assessing fungal biodiversity.

## Introduction

Most of the spatial diversity and distribution of fungi is poorly known (Větrovský et al. 2019). A primary reason for this is because most of it lies below ground, a habitat which has been a mystery for centuries (Kirk et al. 2004). However, the more affordable high-throughput sequencing (HTS) technologies in terms of cost and execution time (Churko et al. 2013) enable researchers to use the metabarcoding technique for their studies; a method that reveals fungal communities in environmental samples, such as soil (Schmidt et al. 2013). This practice has become a major factor in contributing to the research of fungal biodiversity (Nilsson et al. 2018a) and many publications that characterise fungal communities in soil are growing exponentially (Song et al. 2015). Further, contributors are encouraged to share the data generated by the HTS technique in sequence read archives (Wilson et al. 2021, Katz et al. 2021), for which the National Center for Biotechnology Information (NCBI) Sequence Read Archive (SRA) (Leinonen et al. 2010) is widely used. Essentially, this procedure offers a great advantage for acquiring fungal biodiversity knowledge. However, the data provided do not allow direct use by others as a result of being experimental DNA sequence data that are not yet curated and identified.

The MycoDiversity Database User Interface (MDDB-UI) (https://mycodiversity.liacs.nl) we present here exhibits that the data provided by other researchers in the public repositories, namely in the scientific literature (PMC 1988) and metabarcoding studies, can also be used directly for gaining the required information. The accessibility through the MDDB-UI is permitted by the main data elements provided in the Biodiversity Search tool: the taxonomy, the geo-location and the abiotic characteristics of the environment. These key elements are essential to unlock the hidden fungal biodiversity from the public metabarcoding studies. The taxonomy is used to determine to which taxa the genetic barcodes (i.e. markers of DNA sequence information) of the fungal organisms belong. The geo-location is crucial for obtaining spatial information regarding where samples of studies have been collected and to observe where fungal taxa occur. The latter element, the environment, is the contextual aspect relevant to observing under what conditions a fungal community occurs.

The important feature of the MDDB-UI Biodiversity Search tool is the multidimensional structure of these data elements, presented to users in the form of dynamic hierarchical tree views. This method guarantees the direct observation of fungal biodiversity data by using various taxonomic levels. Furthermore, it grants the exploration of fungal distribution data along different spatial terrestrial scales. Moreover, the environment element enables the exploration of how species richness varies under different intervals of conditions, such as acidic ranges in soil (Rousk et al. 2010, Glassman et al. 2017).

The MDDB-UI provides to users the element values for predefined SQL queries. The query results are presented in topological maps and configurable table views, retrievable for further fungal biodiversity investigations and for supporting the evolutionary and ecological research on fungi.

## Background Information

### Fungal biodiversity

Measuring biodiversity over large spatial areas, varying from regions to countries, or a global scale, usually involves combining collections of species observations obtained from environmental space (Jetz et al. 2019). For microorganisms, this is more challenging as it requires more effort to explore and describe what cannot be observed macroscopically. As a consequence, compared to other major groups of eukaryotic organisms, the spatial diversity and distribution of fungi is not fully completed. Most of the Earth’s terrestrial surface is covered by soil, a habitat known to have high levels of fungal biodiversity (Orgiazzi et al. 2016), thus making it an important component to explore. This diversity, however, has remained a ‘black box’ for a long time, until the development of DNA barcoding finally enabled the exploration of these taxa (Seifert 2009). In particular, the application of HTS technologies on these DNA barcodes has transformed our perspective on fungal distribution by enabling the detection of organisms directly from their environments (Tedersoo et al. 2022).

### The MycoDiversity Database and association with public metabarcoding data

Currently, there is a meaningful focus on sharing data (Popkin 2019) and public data repositories have evolved to provide access to data generated by HTS techniques. As a consequence, data belonging to metabarcoding studies conducted in different locations around the globe are available in common open sequence read archives, of which the most commonly used is the Sequence Read Archive (SRA) (Shumway et al. 2009, Leinonen et al. 2010). These repositories undoubtedly contain valuable information to assist research in fungal biodiversity. However, the data provided do not currently allow for direct use. As encountered by Martorelli et al. 2020 and Jurburg et al. 2020 we face two obstacles. The first relates to a classification problem; these archives contain the original raw sequencing data generated by experiments using HTS platforms, which have not yet been curated and assigned to a particular taxon. The second relates to the description of the collected samples of these studies, which is provided in different formats and the annotation is either lacking or described in diverse manners. The development of the MycoDiversity DataBase (MDDB) addressed these two main concerns by classifying and organising the data in a uniform manner. Subsequently, it established the proper associations for linking the curated reference DNA sequence barcodes (i.e. Zero-radius OTUs, commonly known as ZOTUs) (Edgar 2018) to taxonomic scientific names and to the relevant records in public archives. The work described in this paper focuses on unlocking and combining these data through an interactive user interface provided by the MDDB-UI.

### Biodiversity repositories - related work

Unifying collections of species observations has a long history. The digitisation era, in which we are currently living, permits direct access to large-scale species observations. These recorded observations are based on the effort of integrating previous descriptions of human observations, particularly on specimen samples. The Global Biodiversity Information Facility (GBIF) (Holstein 2001, GBIF 2021) is a valuable source for biodiversity assessments and distribution for species from various lineages of the tree of life. The species descriptors contained in different digital collections vary, but GBIF displays high-quality integrated data that originate from the effort to map standard formats and terms to collections of these datasets. The power of this strategy allows an increase in the properties of the metadata and enables the use of standard terms (e.g. reference taxonomic names) and values (e.g. such as time stamps and dates linked to observations and species occurrences). The novelty of this platform also lies in the different data views provided. These include maps for visualising species occurrences and treemaps to display hierarchical data, such as taxonomy. Recently, the GBIF consortium announced the importance of incorporating barcode information (Finstad et al. 2020) as it is relevant for including species detection, based on environmental observations. GBIF has already included a new implementation that allows users to submit HTS data on their platform. However, this feature permits the publication of processed assigned HTS data as taxon occurrences, thus restricting the possibility to trace the original data source, including experimental metadata and the provenance of the sequence barcode representatives.

Another crucial aspect to consider is that data repositories, in general, evolve to adjust to the data they host. Occasionally, these repositories need to be redesigned in order to incorporate different and novel data sources. In line with making data open (OpenScience 2023, Dataverse 2023), important protocols should be imposed for submitting new data by following the data FAIR principles (Wilkinson et al. 2016). Additionally, researchers should be encouraged to incorporate the FAIR4RS software guidelines when developing methods for generating data, as well as database design (Barker et al. 2022). These recommended guidelines should become the standard practices for data submission and availability in the biodiversity domain (Belien et al. 2022, van der Velde et al. 2022). PlutoF platform (Kõljalg et al. 2019) is designed to manage different data types (e.g. ecological data, taxonomic backbones, DNA sequences, specimens, human observations and sample materials), all information that needs consideration and should be integrated while managing biodiversity data.

Regarding fungal biodiversity accessibility obtained from HTS source, considerable work is provided by the GlobalFungi database (Větrovský et al. 2020). GlobalFungi offers direct access to barcoding data obtained from sequence read archives for specific fungal species and genera. Its functionality focuses on providing fungal occurrences on a world map and displaying fungal biodiversity patterns using bioclimatic factors (e.g. mean annual temperature and precipitation) and pH ranges. Like GBIF, this platform highlights the benefits of using controlled terms to increase metadata integration, in particular related to the habitat. It also relies on a uniform methodology for curating HTS data, which aligns with the principles of barcode data integration of MDDB and the approaches of Meiser et al. (2013). While indeed Větrovský et al. (2020) expose the same baseline regarding fungal detection and occurrences from metabarcoding studies, their methodology for curating sample metadata relies on manual methods and the input of the authors of the studies. We provide spatial observations at regional levels, based on automatic methods used for curating the spatial component metadata (Martorelli et al. 2020, QGIS 2023, Geonames 2023). This approach allows us to enhance the multi-dimensional aspects of our searching tool, such as to include the use for observing the community levels. As we rely on the power of linking taxonomic types and persistent identifiers of controlled taxonomic reference names, this has proven to be a great approach for providing insights into multi-fungal groups of species. In the next section, we focus on illustrating the accessibility, the multi-dimensionality and the reliability of data provided by MDDB-UI. These are important features to consider not only for increasing the data reusability, but also for improving the assessments and research in fungal biodiversity.

## System Design

**Figure 1.**
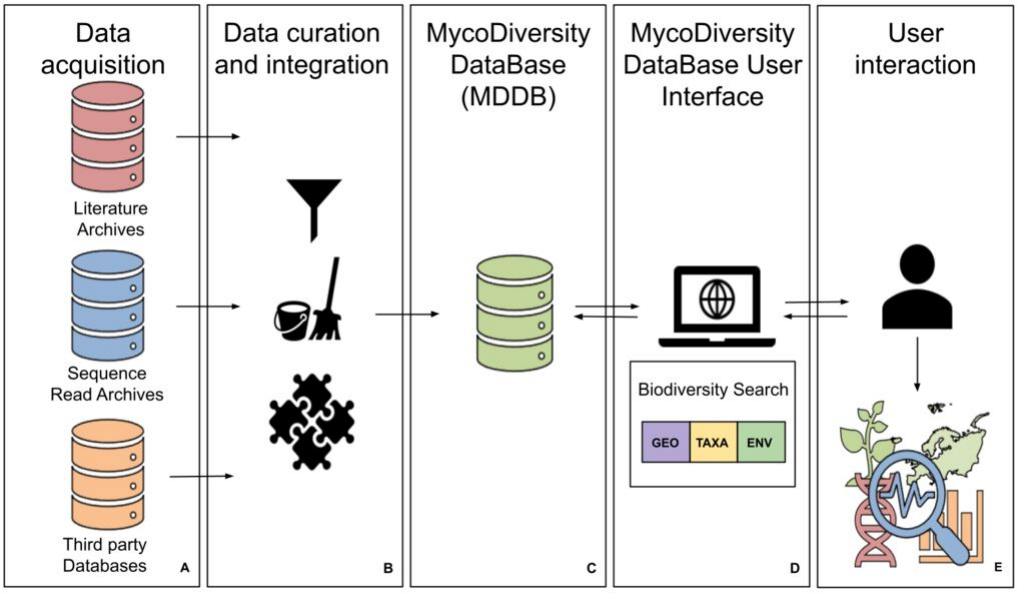
Workflow of the MDDB for the exposure of the front-end Biodiversity Search tool.

The main areas of design criteria for the MDDB-UI system are the conceptual data design, the user interactive design and the technical design. These principles define the mapping of the MDDB back-end to the front-end interactive tools as illustrated in Fig. 1. The first tool we developed and describe in this paper is the Biodiversity Search tool (Fig. 1D). This tool provides the user with direct access to fungal taxa from DNA barcoding sequence data. It is also meaningful to determine where the classified DNA barcode sequence is detected and under what environmental conditions it occurs. This scenario defines the fundamental components that constitute the inference of fungal biodiversity amongst public data collections. The data contained in MDDB (Fig. 1C) rely on the unprocessed and unclassified metabarcoding data acquired from the studies deposited in sequence read archives (Fig. 1A). It is, however, through the curation and the integration with data from reference public databases (Canese and Weis 2013, Nilsson et al. 2018b, Geonames 2021) that we can classify and reveal it as significant barcoding data for use in fungal biodiversity assessments (Fig. 1B). The interaction with the tool provides such meaningful data for assisting users in the research and analysis of fungal biodiversity (Fig. 1E). In the following sections, we exhibit the main criteria of the Biodiversity Search tool of the MDDB-UI.

### Conceptual Data Design

The main conceptual data design criteria of MDDB-UI are the availability, dimensionality and connectivity of the fungal biodiversity components.

#### Availability of the main components

**Figure 2.**
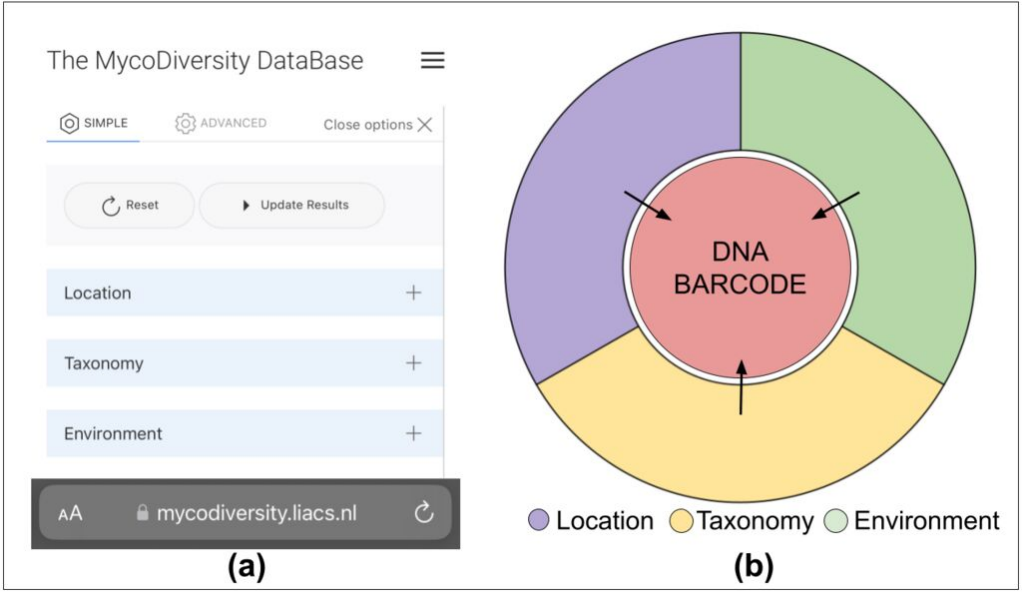
Illustrations of the available elements used for unlocking DNA barcode data. **(a)** Location, Taxonomy and Environment are the key elements of the Biodiversity Search tool made available to the user for initiating the search in MDDB; **(b)** The interaction with one of the elements unlocks and reveals the DNA barcode data element.

The elements of the Biodiversity Search tool displayed to the user are the Location, Taxonomy and Environment. These represent the key components for the user to interact and initiate the search (Fig. 2a). Thus, the user's interaction with one or more of the main elements allows unlocking the connection of the components. It establishes access to the curated DNA barcode data component (Fig. 2b), which is associated with metabarcoding studies.

The taxonomy element utilises the scientific standard names to obtain the DNA barcode information. The interaction with the location element enables observation of where the DNA barcode is collected and the environment is used to inspect under which environmental conditions the DNA barcode has been detected.

#### Dimensionality of the main information components

**Figure 3.**
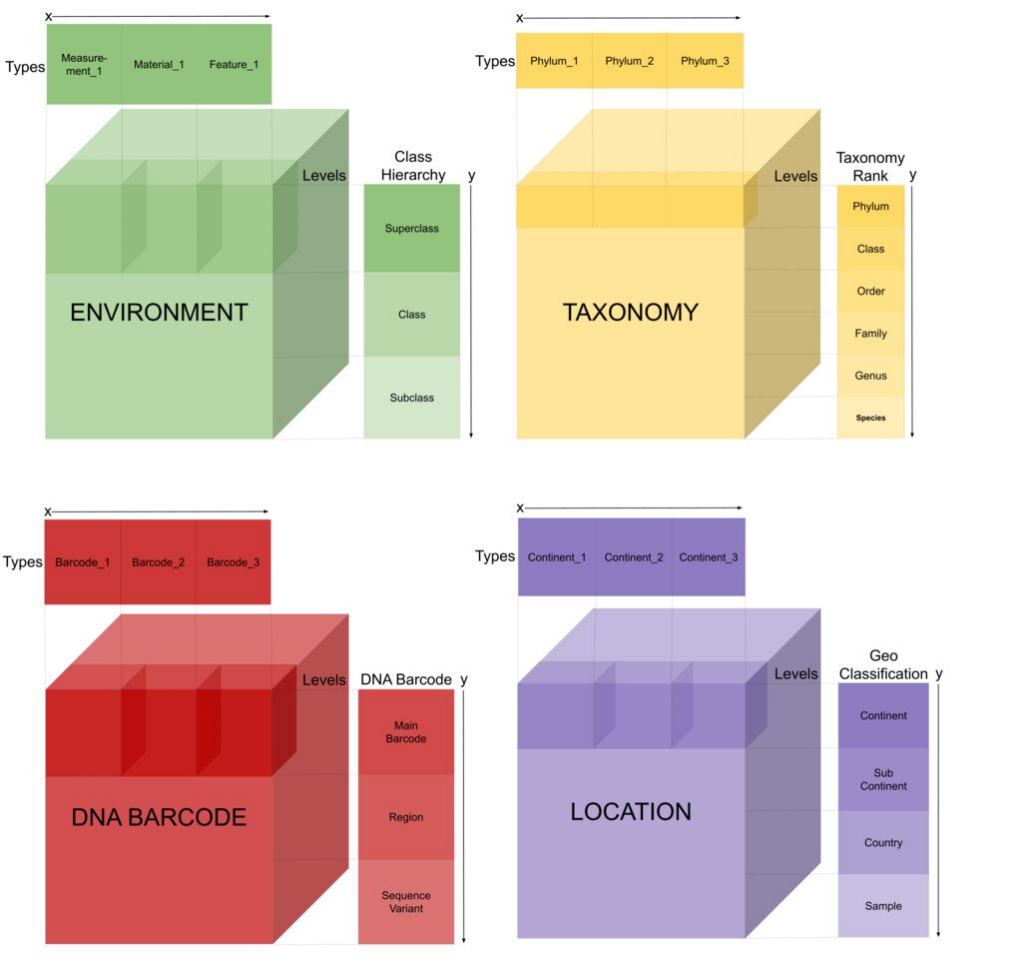
Structure of the main information components of the Biodiversity Search Tool of MDDB-UI is illustrated as cubicle containers.

Due to the complex nature of the data and their multiple dimensions, it is essential that the system supports various data views. The Taxonomy, Location, Environment and the uncovered DNA barcode components are containers of structured information (Fig. 3). For each component, navigation through these structures is permitted, enabling direct access to every group of items (i.e. a highlighted box display for each block of the Fig. 3.x-axis, labelled 'Types') at each hierarchical level (i.e. the Fig. 3.y-axis, labelled 'Levels').

The Taxonomy (Fig. 3.Taxonomy) provides the classification of fungal taxa, based on fungal reference scientific names (Abarenkov et al. 2021). Navigating to the lower levels of this component allows for alternative data aggregations, collecting more defined sections of the taxonomic ranking. It is possible to directly observe either a group of species belonging to a high-level group (e.g. a phylum) or a more targetted group (e.g. family level).

The Location compartment (Fig. 3.Location) follows the same principles as the Taxonomy hierarchy, whereas layers obey the partonomy classification of the geographical area names (Geonames 2021). Using this component enables navigation from a large-scale territorial area (i.e. continental level) to a more refined geospatial region of interest, provided as a specific position where a referenced sample is collected. In this context, we will use the term "plot" for the sample site in a geographical space. The identifiers of the original samples are also associated with their spatial values representing their GPS coordinates.

The DNA Barcode (Fig. 3.DNA-Barcode) also contains organised structured information, as DNA barcodes are specific informative regions of the organisms' genome. Most current metabarcoding studies on fungi that are accessible in sequence archives, provide DNA sequence fragments (i.e. reads) belonging to specific ITS2 or ITS1 segments of the ITS barcode region (Schoch et al. 2012). While not as frequent, other studies also use SSU and LSU (e.g. archive studies SRP015917, ERP010906, SRP042134) as DNA barcodes. It is laborious to identify the type of barcode fragment sequences in SRA studies related to fungi, as this annotation term is rarely provided in the HTS experimental records of these studies (SRA 2022) and is infrequently found in a standard way. One needs to recognise that HTS technologies are also evolving (Ambardar et al. 2016, Amarasinghe et al. 2020). At present, Heeger et al. (2018) show that longer stretches of sequence barcode regions can be sequenced by novel HTS platforms. Given these reasons and considering that MDDB continues to incorporate more studies over time, the DNA barcode container is designed to accommodate the inclusion of the genomic data segments that represent the barcodes used for the various phylogenetic groups of species.

The latter component (Fig. 3.Environment) contains two data types; the Environmental Term and the Environmental Measurement. The Environmental Term value can constitute either a Feature, which is a term used to define the habitat (e.g. biome term) or the Material, that is a physical term that defines the environmental sample collected (e.g. soil). Both these types of descriptors are defined by the Environment Ontology controlled terms (ENVO) (Buttigieg et al. 2013). The Environmental Measurements are quantities, where the levels correspond to intervals of ordinal values (e.g. acidity levels, organic carbon concentration). These values rely on Units Ontology (UO) (Gkoutos et al. 2012) and Ontology of Units of Measure (OM) (Rijgersberg et al. 2013).

#### Connectivity of the components

**Figure 4.**
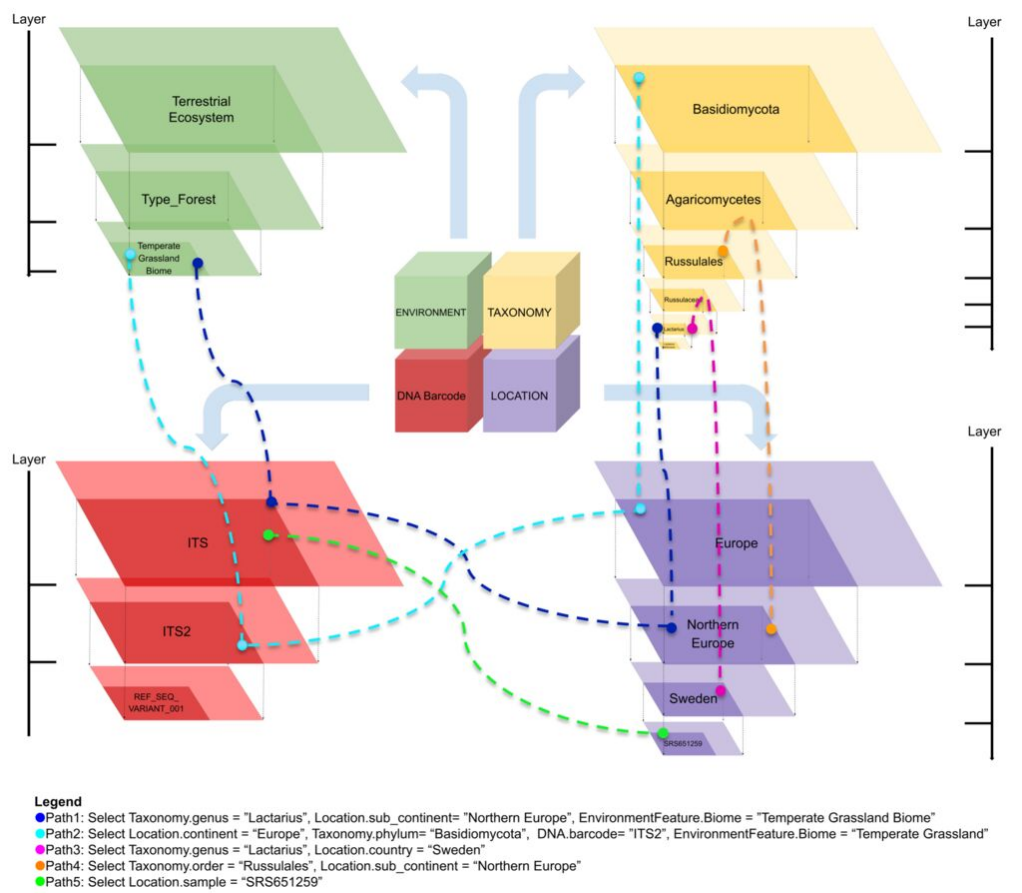
Connection of the items belonging to the main containers.

The relationships amongst the main block containers form journey paths in which terms of the various components and of different levels connect (Fig. 4). Points of these exploration paths originate from any of the initial top-levels (e.g. phylum rank of the Taxonomy, the x-axis) of the main containers and navigation may continue along the different hierarchical levels (i.e. the y-axis). The connection of the terms belonging to the different containers (i.e. the dashed coloured lines) is enabled when one or more of the main elements is initiated during the interaction with the search tool.

For simplicity, only one term for each tree-map level of the main containers is revealed. Path 1 (the dark blue label) exhibits the choice of focusing on a specific taxonomic rank (term genus *Lactarius*) observed at the sub-continental level (term Northern Europe) in a temperate grassland biome context. The connection to the ITS barcode of the DNA barcode container is by default, but the annotation for the type of ITS (i.e. barcode ITS2 or ITS1) is provided. For path 3 (pink label), a more specific selection emerges; the DNA barcode sequence variants of ITS2 belonging to *Lactarius* that have been observed in the samples collected in Sweden, independently of the contextual aspect, are collected. Conversely, path 2 (cyan label) reveals a more generic search of interest, which considers the higher level of taxonomy (Basidiomycota phylum) that has been observed on a broader scale, the continental level (term Europe). This data selection can be sequentially refined, as shown by path 4 (orange), where only the DNA sequence variants belonging to the Russulales order level and detected in countries belonging to northern Europe are selected. Path 5 (the green label) suggests a simple yet a novel, powerful approach for exploring fungal biodiversity data when the Taxonomy component is not initiated. This case demonstrates how to directly observe a community at a small-scale level of diversity (e.g. the selection of a single specific sample), for which all sequence variants can be provided, regardless of their assigned taxonomic reference name.

### User Interface Design

**Figure 5.**
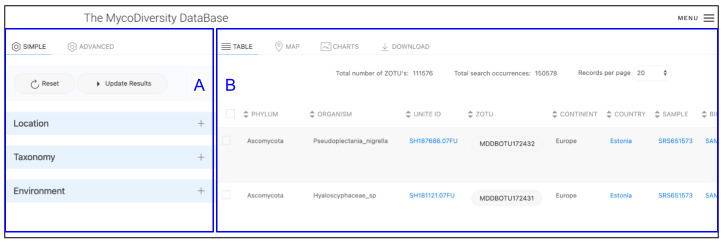
Browser display of Biodiversity Search Tool (A) alongside the Results window display of the query results (B).

The web-based MDDB front-end application for the Biodiversity Search tool is developed using the criteria for user-centred design (Nielsen 1994, Jaspers 2009, Nielsen and Norman 2023). For the design, the conceptual data model (Fig. 3) is mapped to the interaction and interface design (Fig. 5.A). Experts evaluated the initial designs and the Nielsen usability heuristics (Nielsen 2012) guided the process. This method allowed the development of an efficient and clear front-end interface (Fig. 5). Nevertheless, the interface can continue to adapt and improve and, therefore, suggestions from researchers that are integrating the resources in their research workflows are always considered.

#### Usability

The interface uses the graphical basics and widgets provided by modern browsers and APIs. In graphical user interfaces, the elements such as icons and pictograms are included that have a clear perceived affordance (Norman 1988). Moreover, researchers are accustomed to these standard visual elements and their perceived affordance. Importantly, the widgets in the design are placed corresponding to the logical visual flow of the system. A colour coding of the page facilitates quick separation of relevant parts in the interface and the selection items used for the elements. Likewise, the intuitive iconography guidelines help users expand hierarchical data and perform tasks. The visibility, or feedback, from the interactive elements is immediate. The group of researchers we engage include mycologists, taxonomists, evolutionary biologists, ecologists, as well as field amateurs, climate change scientists, conservationists, engineers, and researchers from other domains, along with first-time explorers, like students. For this reason, the interface provides familiar and intuitive visual icons and text descriptors to enhance usability for a broad range of users. The philosophy behind MDDB is based on the principle of learning by doing. The logic of presenting results is optimised for memorability.

#### Evaluations and Consistency

The relation of the usability aspects (Norman 1988, Nielsen 2012, Harrison et al. 2013) and the design are given in Table 1. The design considerations are written after expert evaluation and user consultation (Kurniawan 2004). New elements can be added to the interface after evaluating them with the same usability heuristics.

## Technical Design and Implementation

In this section, we describe the technical design criteria for shaping the MDDB-UI (https://mycodiversity.liacs.nl). The criteria cover the accessibility of the data, the visual presentation of results, data retrieval and the performance of our system.

### Open Access

**Figure 6.**
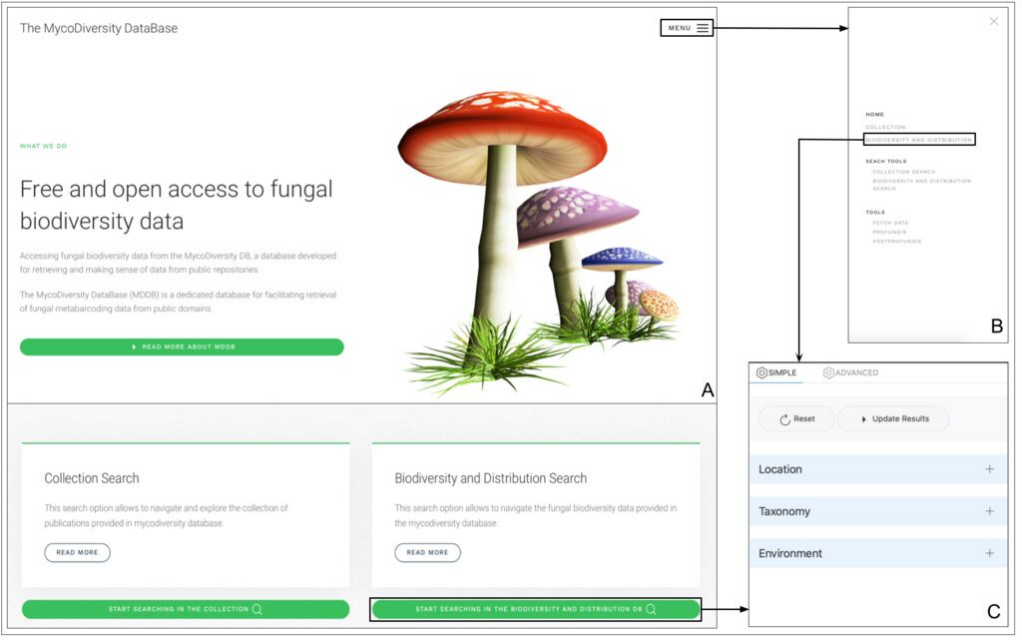
Main web page design of the MDDB interface showing redirection of the Biodiversity Search Tool from the home page and Menu window.

The MDDB-UI is a browser front-end that provides free open access to information stored in MDDB. The home main page (Fig. 6) provides direct access to the Biodiversity Search Tool (Fig. 6C).

On the MENU option of the home page (Fig. 6B), we provide links to methods developed for collecting and processing the information from the studies. These repositories are made available to enhance data reusability (Github repository 2021, GitHub repository 2021) and improve data reproducibility (Github repository 2020). The front-end, defined by the main page, headers, footers and the menu, is developed using Joomla, a content management system (CMS) (Joomla 2021) (version 3.10). Whereas, access to the data is enabled by the Microsoft Open Database Connectivity (ODBC) driver (Engel et al. 2021), through which the web server connects to the database server hosting the monetDB MDDB.

### Selection of data: queries

**Figure 7.**
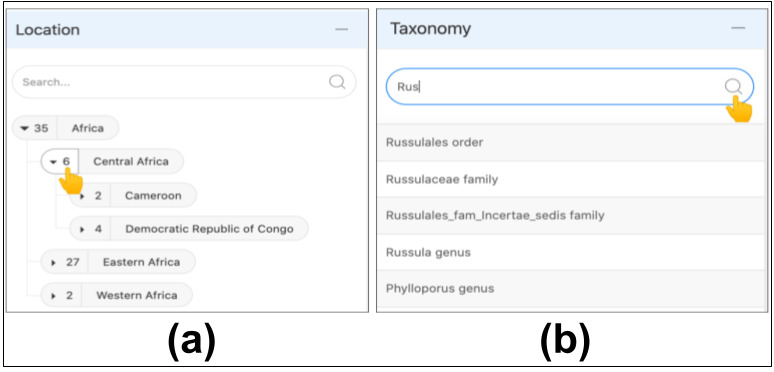
Navigation of items of the Location and Taxonomy compartments. **a** Display of a tree view selection. One subclass (i.e. subcontinent) of the continent 'Africa' is selected. **b** The Taxonomy autocomplete search. At least three characters are needed to initiate the filter and suggestions containing the characters are displayed.

The data access to MDDB is granted in an intuitive manner, considering both the nature of the data and the diversity of the users. The usability attributes (Table 1) and the visibility principle, defined in the section User Interface Design, guided the implementation of the search display functions of the Biodiversity tool. As the Location and Taxonomy components contain hierarchical data, the accordion content search allows viewing targetted terms of the tree. The numerical values next to the names of the layers represent the number of items contained in each layer. Clicking on the value allows expansion of the tree to reveal lower levels (i.e. subclasses) (Fig. 7a). Users can employ the search box to jump directly to items of interest (Fig. 7b). The tree is primarily used for exploration, whereas the autocomplete search option is more useful for directly accessing known taxa.

**Figure 8.**
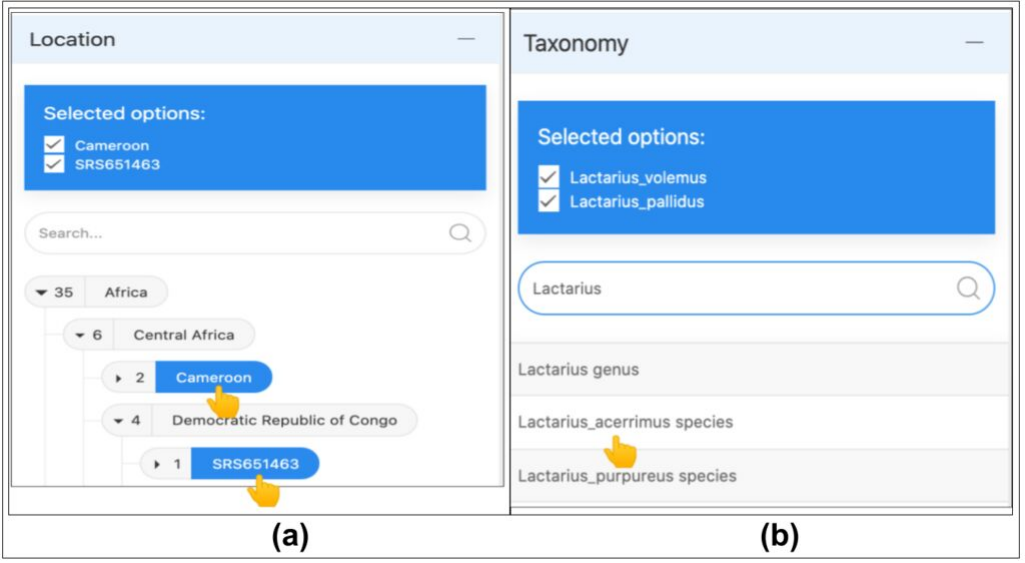
Query construction using the search tool. (a) The Location subclasses of the continent 'Africa' are displayed. The country and the sample site, shown as checked selected terms, are the items used for the query; (b) The Taxonomy autocomplete selection: illustration of focusing on a specific taxa group. The autocomplete selection is useful for directly accessing the lower levels of the taxonomic tree.

A value is selected when the user clicks on an item and it is displayed as a checkbox (Fig. 8a). According to the effectiveness attribute in Table 1, all the children of the layer are included for a selected item. The same method applies to the autocomplete search filter box; the selected item is appended in the list displayed above the search (Fig. 8b). The deselection of the items is allowed by clicking on the item name or on the checkbox.

**Figure 9.**
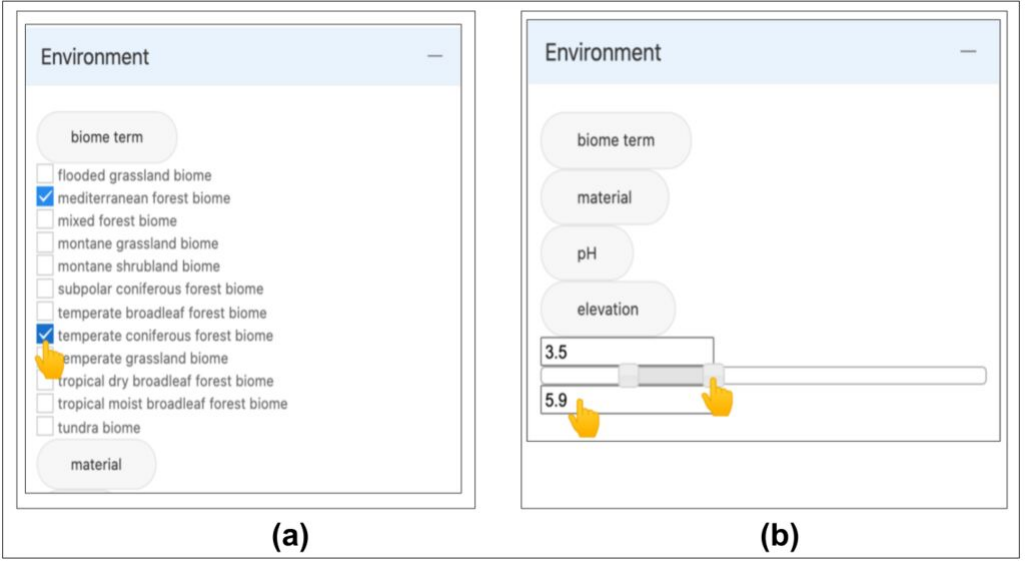
Environment filter types. **a** The ENVO terms 'mediterranean forest' and 'temperate coniferous' biomes are selected; **b** Range slider used for filtering soil samples with pH values in the range of 3.5 to 5.9.

The Environment selection provides environmental terms used to describe habitats and ranges of numerical values. The selected items (Fig. 9a) belong to a controlled list of environmental feature terms and are displayed with checkboxes. Measurement values are controlled through range sliders (Fig. 9b). To increase the accuracy with which users achieve their task, the values selected are controlled and corrected if they do not fall within the proper unit range of the measurement (e.g. pH values >14). The latter choice is based on the fact that most fungal community environmental studies and fungal research investigations involve comparing species richness and diversity across different measurement ranges (Geml et al. 2014, Carrino-Kyker et al. 2016, Liu et al. 2018). We intend to implement the Environment features to follow the hierarchical strategy displayed for the Location and Taxonomy elements, as we defined in our design (section Conceptual Design). When the user is satisfied with the data selection, the 'Update Results' button provides the results in the adjacent window. The 'Reset' button allows users to cancel the data selection (Fig. 6C).

The search implementations are embedded in iframes and use PHP (version 7.4.0) (PHP 2021) and the UIkit (version 3.7.5), a PHP JavaScript framework (UIkit 2021). The language HTML5 (HTML 2021) structures the interface web pages, while JavaScript (Javascript 2021) is used for the expansion of the layers and the JQuery libraries (jQuery 2021) support HTML manipulation.

### Presentation of results: multiple data views

**Figure 10.**
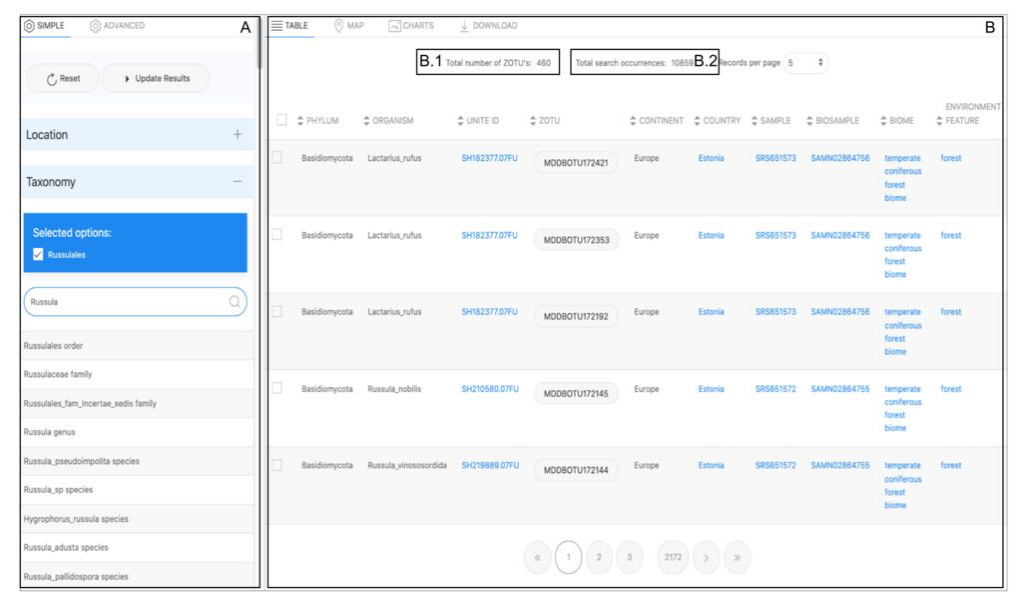
Illustration of the data table view alongside the Biodiversity Search tool. Most of the attributes displayed redirect to the integrated reference public records.

The query results appear in the dynamic output window on Table Tab and the Map Tab (Fig. 10B). Both tabs initialise when the 'Update Results' button of the Search Tool (Fig. 10A) is selected. A JavaScript function controls switching on the data tabs.

#### Table view

The table view is the common method for displaying a dataset output based on a query. Each row of the results shown on the table corresponds to a unique record of a DNA sequence variant hit (i.e. ZOTU) detected from a selection of the filtering options. This hit represents the occurrence and its attributes display the annotation associated with the other components (Fig. 10).

Most attributes in the table appear as hyperlink values, which redirect to URLs, such as the corresponding mappings to the reference taxonomic name or to the provenance sample it originates from (e.g. NCBI BioSample record). These are reliable standards and data sources that follow the FAIR findability data principle (Wilkinson et al. 2016, Stall et al. 2019). These results provide data credibility and allow for extending relations to this knowledge domain. Above the output table display of the Table tab, two important values are displayed. The Total number of ZOTUs (Fig. 10B1) represents the total number of distinct ZOTU sequences obtained as a result of the query. The Total search occurrences (Fig. 10B2) is the record count of ZOTU sequences detected hits for the condition chosen by the user.

#### Map view

**Figure 11.**
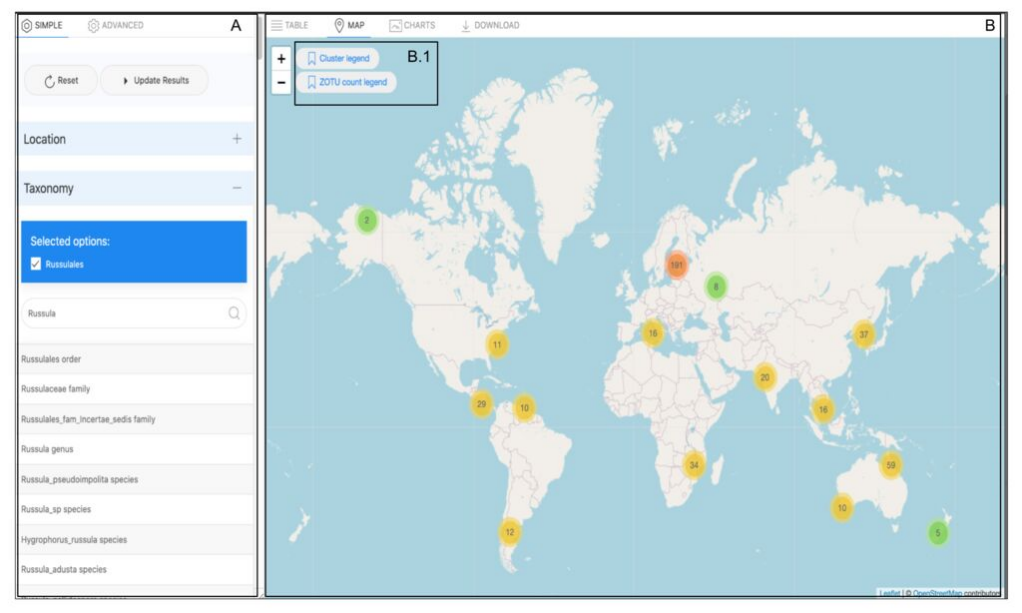
Illustration of the data map view alongside the Biodiversity Search tool (Fig 11A). The map view is initiated by clicking on the Map Tab (Fig 11B). Two legends displayed on the map guide the visualisation (Fig 11B1).

The map view is an associated function of the table view and initiates when the user clicks the Map Tab button (Fig. 11B).

**Figure 12.**
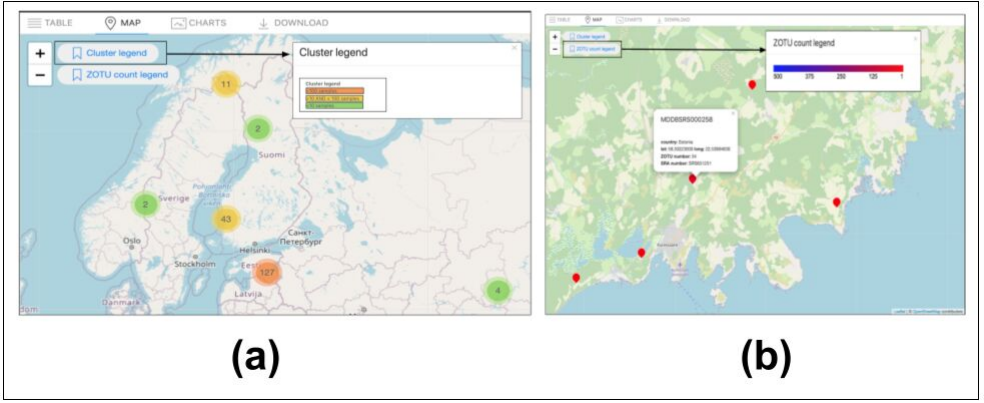
Map view example of a geographical region. (a) Number of sample representation of the map view; (b) Sequence variant diversity (ZOTU richness) display for one soil plot (sample SRS651251).

All samples stored in the MDDB include a geographical location specified as a decimal numerical value (GPS) (Sinnige and Polen 2020). Two important measurements derive from the occurrence query display of the table view; these are visible in the legend, represented by two different colour gradients. These gradients provide the dimensionality of the data visualised on the map (Fig. 11B1). The first measurement relates to the physical location and matches the number of clustered samples where a DNA sequence has been detected (Fig. 12a). This measurement utilises an orange to yellow to green gradient to represent the quantity of samples in a spatial area, with orange indicating an area denser with samples. The second measurement connects to taxonomic labelling, inspecting the fungal groups. Through interaction with the map, this measurement grants the observation of the sequence richness within a community comprising the sample (Fig. 12b). The values of this richness appear through a dark purple to red gradient, with darker colours indicating richer communities.

This latter information is perceived on the interactive map, where the marker icon embedded over each sample indicates the amount of DNA barcode sequences, represented as ZOTU numbers, observed in the original sample plot. The total numbers of plots and ZOTUs shown on the map reflect the aggregated results of the occurrence hits displayed on the table view (Fig. 10B). The interactive map view is built in JavaScript by using the Leaflet package (version 1.7.1) (Leaflet 2021). Using controlled terms to describe data enhances data aggregation and extends data visualisation functionalities. Our front-end design considers additional implementations of query results, such as dynamic charts, to facilitate the data aggregation display. This Tab, as shown in Fig 10, is only for a User Interface Design display.

#### Data Download

**Figure 13.**
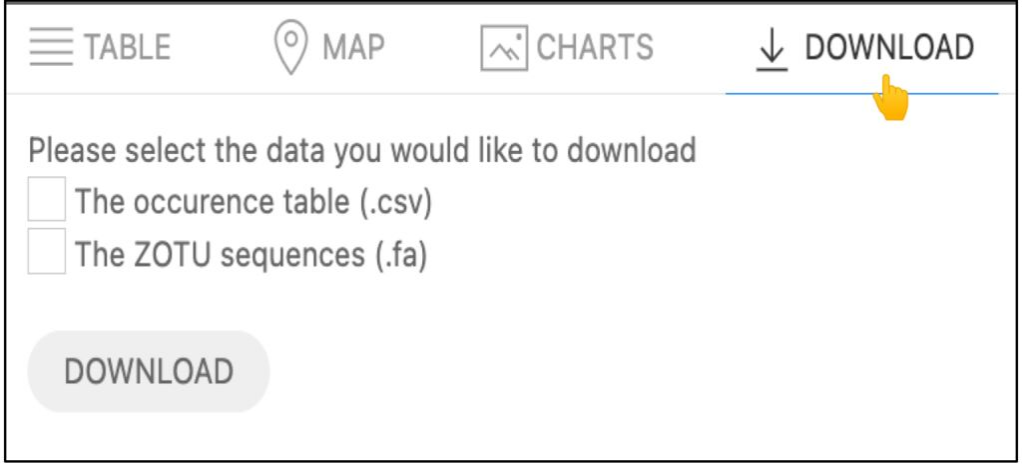
Download Tab window displays the options for retrieving the selected data.

The data selected by the Biodiversity Search tool can be downloaded by selecting the Download Tab (Fig. 13) displayed above the Output Result window, as shown in Fig. 10.

The Download tab provides two options for obtaining the selected data: either the occurrence metadata or the set of distinct ZOTUs sequences detected by the selection. The total occurrence data, indicated by the number of occurrences records displayed in Fig. 10B2, can be downloaded in a CSV format and includes contextual information about the data selection. This information includes the complete metadata related to the location, taxonomy, environment and its associated reliable data standards. The sequence variants of interest, corresponding to the unique count of ZOTU sequences (Fig. 10B1), can be downloaded separately in a FASTA format file. The group list in the FASTA file contains distinct DNA sequences found in the associated site plots or within the chosen taxonomic groups and represent the ZOTU richness. Alternatively, individual ZOTUs from each record of the table view (IV attribute column of Fig. 10B) can be downloaded in individual FASTA files. The user can then remap the occurrence records to the FASTA file as both files refer and contain ZOTUs identifiers, provided by using the MycoDiversity DataBase unique *refsequence_pk* labels references in the file's headers. It is part of our next implementations to make these primary keys be persistent identifiers (i.e. PIDs).

#### Scalability of the MDDB system

The scalability requirement takes into account the nature and the volume of the data. The data comprise large datasets of DNA sequence barcodes and annotations from literature and metabarcoding studies. Extensive queries (Martorelli et al. 2020) that incorporate aggregations of DNA sequence data into hierarchical data and geographical spatial components are efficiently executed by the large-scale processing architecture of the MonetDB system (Boncz et al. 2008, Cijvat et al. 2015). Users can experience this by accessing the large data volumes provided by the MDDB Biodiversity search tool. The system's remarkable response in accessing the data and providing the results to the end-user enhances both the utility and satisfaction with the tool.

## Biodiversity search use case: *Fusarium* in regions of Asia

We present a comprehensive scenario that demonstrates a potential use case for interacting with the Biodiversity search tool. This interaction enables access to and acquisition of informative data from the MDDB for a specific fungal biodiversity investigation. The learnability and efficiency principles covered in the Usability subsection of User Interface Design, are useful for extending the utility of the interactive tool for further fungal biodiversity inspections.

Bananas are the world’s fourth most important food crop after rice, wheat and maize and are grown in more than 130 countries (Voora et al. 2020). Asia is the largest banana producing region and Southeast Asia is known as the primary diversity centre of bananas (Debnath et al. 2019). There is a long history of banana cultivation in this region, with the main focus on selecting more fruitful varieties. During the initial banana export trades of the late 19^th^ century, Panama disease was first reported. This disease is the common name for the Fusarium genus (Ploetz 2015). (Stover and Simmonds 1987) considered Fusarium to be one of the most destructive of all plant diseases, significantly affecting the production of bananas. Studies (Dita et al. 2018, Bubici et al. 2019, Nadiah Jamil et al. 2020) describe potential resistant banana crops by applying biological, chemical and cultural methods to manage the reduction of the pathogen. Most of these methods can be invasive and labour-intensive. However, there is still the need to better understand and study the factors responsible for disease development. For example, abiotic factors can also influence disease intensity (Dita et al. 2018), particularly the pH of the soil. Evidence shows higher levels of the pathogen in banana crops, with Fusarium disease being most severe in those with lower pH values (Domínguez et al. 2001, Nasir et al. 2003, Deltour et al. 2017, Pegg et al. 2019). Nevertheless, there is still the lack of comparative analysis that allows a better understanding of how diverse abiotic factors, such as pH levels, may influence infections.

**Figure 14.**
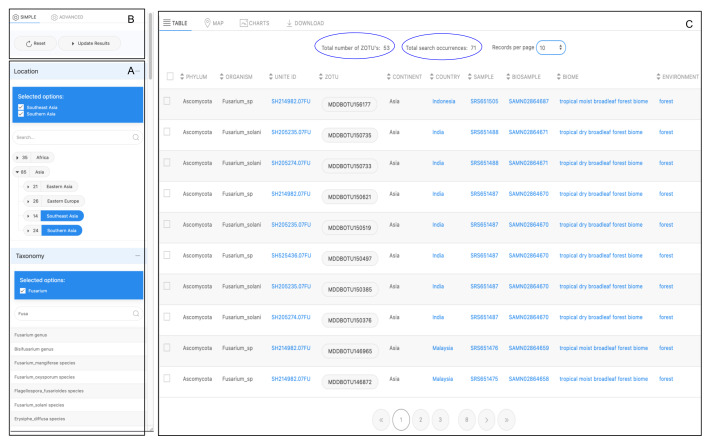
Window A. shows the Location and Taxonomy Search terms selected by the user and displayed by the system. When the user is satisfied with the search selection, the Update Result button is clicked (Window B). Window C presents the Table view of the immediate results of the submitted query.

Here, we illustrate how the Biodiversity search tool can be used to collect data from other studies to enhance the investigation of environmental factors that may influence the occurrence and diversity of Fusarium species. The Location and Taxonomy components are initiated for this use case. For the Location, the tree view is preferably used, as this permits visualisation of the available subclasses of Asia, defined as sub-continents. These include two regions: southern Asia and south-eastern Asia. For the Taxonomy selection, the user focuses on a specific group, with the taxa known. Thus, the autocomplete search box is used to directly obtain the term Fusarium (Fig. 14A). The top hits of the taxonomic names displayed to the user also provide the ranking level, which helps to know the taxonomic level being referred to. The Result button is pressed (Fig. 14B) and the output query result is directly displayed in the Table view (Fig. 14C).

**Figure 15.**
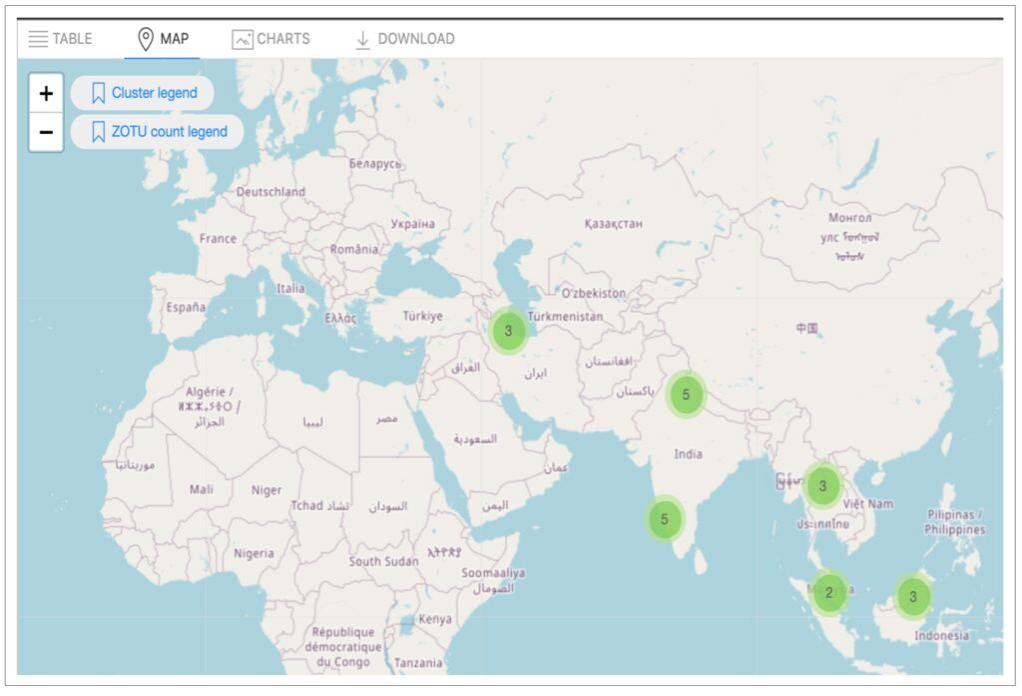
Illustration of the Map View of the 21 samples obtained from the query result, corresponding to the user selection region of interest.

**Figure 16.**
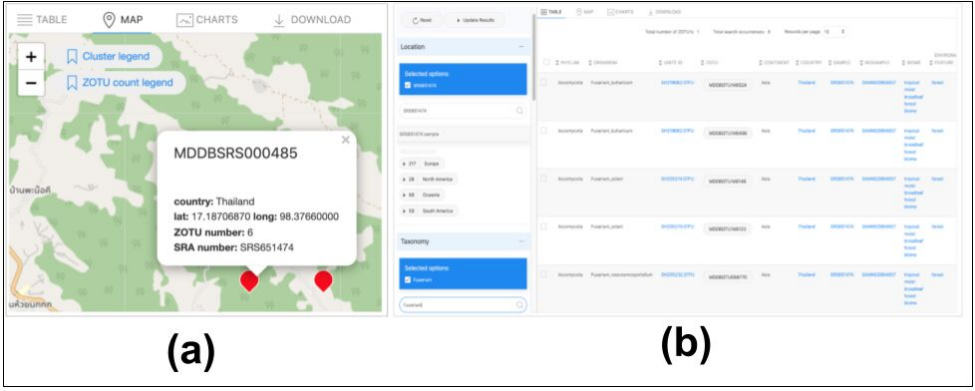
Example of occurrence data display using the map view and data table view simultaneously. **a.** Map view: The markup of plot MDDBSRS000485 (SRA Sample ID SRS651474) displaying the number of ZOTUs observed; **b.** Table View: The six records belonging to the Fusarium genus associated with the SRA Sample SRS651474.

The query result (displayed in Appendix) of this use case returns 71 ZOTU records that correspond to a distinct set of 53 Fusarium sequence variants hits detected for the two regions of Asia. The interactive map displayed on the Map Tab option displays the number of sequence variants for each sample (Fig. 15). The number of sequences is reasonable as the search is refined to a very low taxonomic rank (i.e. genus level). To validate the sequence richness (i.e. number of ZOTUs variants) for one sample, we replicate the search for a single sample hit from the data selected. For example, Fig. 16a exhibits the reference NCBI Sample SRS651474 containing six unique ZOTU sequences belonging to the Fusarium genus group. These are simultaneously provided in the Table view (Fig. 16b).

The purpose of the use case is specifically illustrative, displaying the usability and utility of the tool. The scenario illustrates that it can provide informative below-ground specific regional information, such as ZOTU diversity for a targetted fungal group and an important characteristic of the habitat, acidity, which can be measured. This valuable information should be included to predict Fusarium richness for further sampling and to build upon ecological assessments that associate the above-ground layer. This helps prevent the invasion of the disease on future crop plots.

The Biodiversity Search tool makes an important contribution to scientific research in agriculture. It allows for the observation of fungal communities where Fusarium has been detected, based on reliable existing published data, as described in the design criteria in the Connectivity of the Components subsection of the Conceptual Data Design. Studies analysing Fusarium soils have identified other fungal genera in these soils that play a key role in suppressing Fusarium (El-Baky and Amara 2021). In general, observing groups within microbe communities permits the detection of specific antagonists that can be correlated with the disease suppression (Bubici et al. 2019). MDDB enables the observation of community levels in samples, rather than focusing only on specific fungal groups. This approach can significantly support farmers and community organisations in controlling diseases that invade their crops. For example, farmers could choose to cultivate their new banana crops in fields where antagonists of Fusarium are present in the soils, as observed within these communities.

## Discussion

The scope of this work is to illustrate the importance of the MDDB-UI in assisting research on fungal biodiversity. The most important contributions described in this manuscript are the accessibility, dimensionality of the system and the reliability of the data. These aspects have defined the design and implementation of supportive tools that enhance the use of informative data, which is undisclosed in public metabarcoding studies.

### Accessibility and Dimensionality

The main components of the Biodiversity Search tool provide access to metabarcoding data from published studies. The dimensionality is a unique feature of the MDDB system. Compared to other related systems, as presented in the "Biodiversity Repositories - Related Work" section, it permits the estimation of fungal biodiversity on different levels. The tool allows direct exploration and selection of data from a broader scale (e.g. major phylum group, subcontinental level) to a more targetted taxonomic level (e.g. genus level) and to specific small geographical areas of interest (e.g. one plot sample). The incorporation of samples in the Location compartment allows observation at a local community level. For this case, initiating the Taxonomy is not necessary, which is a powerful approach to access the unique list of unclassified sequence data. It enables direct observation of unique ZOTUs diversity in individual site plots. This novel way of accessing data can be useful for building datasets applied for comparative analysis on community levels and investigating ecological patterns of specific fungal associations. Access to environmental measurements allows observation of DNA sequence occurrences and ZOTU richness amongst different ranges (e.g. elevation), independently of the geographical names of the location where samples are collected. Our primary goal is to permit users to select measurements, such as areas of spatial ranges and observe biodiversity patterns, based on these data selections. The values of the aggregation function used in the queries for associating the different levels of the components and for the connection amongst the different components provides various graphical data views. As observed in the "Presentation of Results: Map View" section, the multiple-layered visualisation shown on the map view displays both the occurrence and the richness of the DNA sequence variants associated with Fungi. Instead of using double-coloured ranges, an extension of this implementation is to use size or height of the plot to represent the measurement quantity.

### Reliability

The multidimensionality of the components of the Biodiversity Tool is reliable as it originates from standard classified sources: currently from the Unite database used for taxonomy, Geonames classification for location and the controlled ontological terms for habitat. The values of these reliable references, incorporated into reference study sources, are provided in the MDDB-UI in the Table View via their persistent identifiers and URLs. Reliability is also achieved through provenance provided by the redirection of the metabarcoding study sources' records. This data provenance, obtained from third parties and metabarcoding studies, is not observed in the tools provided in the "Biodiversity Repositories - Related Work" section. Nevertheless, it is a priority to continue following the principles that lead to data FAIRness (Stall et al. 2019). Another way we increase the reliability of data accessible in MDDB is by providing the methods and descriptions we built to reproduce the processed sequence information (ZOTUs) and the pipelines that can be reused to generate the FASTA sequence files (Github repository 2020).

Additionally, the Literature Search Tool (Martorelli et al. 2023) which we are implementing, is designed to provide comprehensive information from scientific open manuscripts. It includes contributions from researchers and organisations involved in fungal biodiversity metabarcoding studies. This tool will offer direct access to data provenance and detailed descriptions from manuscripts, including terms related to HTS tools and experimental parameters (e.g. HTS platform, instrument settings, primer names), as well as study sources such as metagenomic data submissions (Courtot et al. 2021, FAIR 2023). These terms are essential for refining searches beyond the metadata in sequence data archives. A reference classification of sequence barcodes for organism identification is still lacking in literature. The MDDB barcode sequence compartment is designed to include structured sequence variants, facilitating the integration of the variety of sequence data from studies, including those using novel HTS methods.

Lastly, to increase the meaningfulness of data and of its views, there is a need to include more abiotic factors constituting the contextual aspect of the habitat. These measurements, in their proper standard annotated form and with relevant third-parties incorporation (e.g. bioclimate factors), will extend the use of the relevant factors for investigating the drivers of biodiversity patterns.

## Conclusion and future perspectives

The MDDB interface is an open system for incorporating search tools and graphical data views to interpret hidden biodiversity in public metabarcoding studies. The biodiversity search tool presented in this work provides the dimensions of Taxonomy, Location and Environment for accessing public metabarcoding data. This will expand the interpretation of fungal biodiversity assessments across topographical regions and habitats, providing valuable insights. Additionally, it permits the direct observation of fungal biodiversity at different taxonomic and geographical levels and the exploration of fungal distribution on a visual map.

The use case presented in this work provides an example of obtaining meaningful data for biodiversity research to assist social communities in making data-driven sustainability decisions. Our system is based on controlled, standardised terms which, in combination with automatic cleaning tools, guarantees the quality of the biodiversity data.

One area of future research is to enhance the user interface by developing semantic parsers, based on Large Language Models (LLM) to translate natural language into executable SQL queries. Additionally, we will extend the collection of graphical data views supported by the user interface. Finally, we will study the requirements of biodiversity communities outside the fungal domain to enable a wider application of the MDDB system.

## Appendix: Query and data selection used for the biodiversity use case

Query for generating the results displayed in the Table and Map view of the MDDB-UI. In this case, the components initiated are the geo-location and the taxonomy (Table 2).

SELECT DISTINCT S.sample_pk AS Plot, S.sra_sample, S.country_parent AS SubContinent, S.country_geoname_pref_en AS Country, S.pH, S.elevation, RTDB.genus_name AS ZOTU_Genus, ABS(S.sample_lat_dec) AS Lat, ABS(S.lat_corrected_value_i) AS LatCor, count(C.refsequence_pk) as ZOTURep, count(distinct RTDB.sh_unite_id) as UniteRepFROM Sample as S, Contain as C, RefSequence as RS, AssignTaxa as AT, RefTaxonomicDB as RTDB WHERE RTDB.kingdom_name LIKE 'Fungi\%' AND RTDB.genus_name LIKE 'Fusarium%' AND RTDB.refsequence_taxonomic_pk = AT.refsequence_taxonomic_pk AND AT.refsequence_pk = RS.refsequence_pk AND RS.refsequence_pk = C.refsequence_pk AND C.sample_pk = S.sample_pk AND S.country_parent IN ('Southern Asia', 'Southeast Asia') GROUP BY S.sample_pk, S.country_geoname_pref_en, RTDB.genus_name, S.sample_lat_dec, S.lat_corrected_value_i ORDER BY ZOTURep DESC, UniteRep DESC

## Figures and Tables

**Table 1.** Usability attributes used for the User Interaction design of the Biodiversity search tool.

Usability attribute	Biodiversity search tool UI design choices
*Learnability* First impression with a system and learning how to use a system as quickly and as easily as possible.	The search tool window in Fig. 5A provides intuitive functional data access and a rapid dynamic view for interacting with the results (Fig. 5B). The output results window offers table and map views and displays icons that are universally recognisable. These choices emphasise the “learn-by-doing” concept, where users can effortlessly grasp the fungal biodiversity navigation.
*Efficiency* How efficiently and smoothly the user performs a task.	Efficient interaction with the data is achieved through exploration of the dynamic tree. Terms of interest are suggested in a free search box (Fig. 5A), enhancing user productivity and facilitating navigation of the hierarchical data structure. Experts using standard terms for their predefined queries find this method efficient for locating terms and ensuring consistent results. This aspect aligns with leading to the interoperability principle of FAIR.
*Effectiveness* How a user can complete a task with a high degree of accuracy.	Error reduction is the design criterion applied to aid in effectively controlling the construction of predefined queries. The implementation choice is controlled by the autocomplete function of the free text search box and the use of controlled terms that belong to dictionaries of reference names. This solution ensures precision and accuracy in data retrieval.
*Satisfaction* How a system influences user motivation and effectiveness of use.	For the minimalistic and functional design, we chose blue and white colours because they are aesthetically pleasing. These colours help segment the screen quickly, supporting visual search and focusing attention to the main elements. Likewise, the choice of colours enhances satisfaction by aiding users in identifying different functions. We have also used the “TAB” view to increase visibility and simplify the views.

**Table 2.** Query output view containing the informative data distributed in the table view and map view. The table displays the aggregation of ZOTUs (column attribute ZOTU) for each sample (column attribute MDDB_Plot), based on the user's selection.

**MDDB_Plot**	**pH**	**SRA_Sample**	**Subcontinent**	**Genus**	**ZOTU**	**Unite**
MDDBSRS000484	5.78	SRS651472	Southeast Asia	Fusarium	11	5
MDDBSRS000334	5.39	SRS651487	Southern Asia	Fusarium	8	6
MDDBSRS000328	5.46	SRS651488	Southern Asia	Fusarium	6	5
MDDBSRS000332	4.79	SRS651279	Southern Asia	Fusarium	6	5
MDDBSRS000341	6.76	SRS651332	Southern Asia	Fusarium	6	4
MDDBSRS000485	6.58	SRS651474	Southeast Asia	Fusarium	6	4
MDDBSRS000329	5.25	SRS651277	Southern Asia	Fusarium	4	4
MDDBSRS000333	4.74	SRS651473	Southern Asia	Fusarium	3	3
MDDBSRS000498	5.8	SRS651469	Southern Asia	Fusarium	3	2
MDDBSRS000322	5	SRS651275	Southern Asia	Fusarium	2	2
MDDBSRS000327	4.91	SRS651278	Southern Asia	Fusarium	2	2
MDDBSRS000331	5.07	SRS651276	Southern Asia	Fusarium	2	2
MDDBSRS000338	7.08	SRS651338	Southern Asia	Fusarium	2	2
MDDBSRS000378	3.47	SRS651476	Southeast Asia	Fusarium	2	2
MDDBSRS000379	3.69	SRS651249	Southeast Asia	Fusarium	2	2
MDDBSRS000326	5.64	SRS651485	Southern Asia	Fusarium	1	1
MDDBSRS000330	5.15	SRS651274	Southern Asia	Fusarium	1	1
MDDBSRS000335	3.38	SRS651505	Southern Asia	Fusarium	1	1
MDDBSRS000337	6.49	SRS651331	Southern Asia	Fusarium	1	1
MDDBSRS000374	2.6	SRS651247	Southeast Asia	Fusarium	1	1
MDDBSRS000377	3.5	SRS651475	Southeast Asia	Fusarium	1	1
Count		21			71	
